# Gender Differences in Circulating TFAM Levels Are Associated with Functional Impairment and Sarcopenia

**DOI:** 10.3390/biomedicines14051146

**Published:** 2026-05-18

**Authors:** Olga Laosa, Aitor Carretero, Leocadio Rodríguez-Mañas, Jose Antonio Carnicero, José Viña, Maria Carmen Gómez-Cabrera, Javier Angulo, Francisco José García-García, Mariam El Assar

**Affiliations:** 1 Fundación para la Investigación Biomédica del Hospital de Getafe, 28905 Getafe, Spain; olga.laosa@salud.madrid.org (O.L.); lobouc3m@hotmail.com (J.A.C.); 2 Centro de Investigación Biomédica en Red de Fragilidad y Envejecimiento Saludable (CIBERFES), Instituto de Salud Carlos III, 28029 Madrid, Spain; acarretero@incliva.es (A.C.); leocadio.rodriguez@salud.madrid.org (L.R.-M.); jose.vina@uv.es (J.V.); carmen.gomez@uv.es (M.C.G.-C.); javier.angulo@hrc.es (J.A.); franjogarcia@telefonica.net (F.J.G.-G.); 3 Instituto de Investigación IdiPaz, 28029 Madrid, Spain; 4 Freshage Research Group, Department of Physiology, Faculty of Medicine, University of Valencia and Fundación Investigación Hospital Clínico Universitario/INCLIVA, 46010 Valencia, Spain; 5 Servicio de Geriatría, Hospital de Getafe, 28905 Getafe, Spain; 6 Servicio de Histología-Investigación, Unidad de Investigación Traslacional en Cardiología-IRYCIS/UFV, Hospital Universitario Ramón y Cajal, 28034 Madrid, Spain; 7 Servicio de Geriatría, Hospital Virgen del Valle, Complejo Hospitalario de Toledo, 45005 Toledo, Spain

**Keywords:** TFAM, aging, functional decline, sarcopenia, sex-stratification

## Abstract

**Background/Objectives**: Mitochondrial dysfunction contributes to age-related muscle decline. Mitochondrial transcription factor A (TFAM), a key regulator of mitochondrial biogenesis, may serve as a marker of chronic inflammation and cellular stress when released into circulation following mitochondrial damage. However, its association with functional impairment and sarcopenia, particularly across sexes, remains poorly addressed. The objective was to examine the association between circulating TFAM levels, physical function, and sarcopenia in older adults, considering sex-specific differences. **Methods**: This study included 989 community-dwelling older adults (mean age 75.4 years; 45.6% men) from the Toledo Study for Healthy Aging. Plasma TFAM was measured by ELISA. Functional status was assessed with the frailty trait scale-5 (FTS-5). Sarcopenia was defined by established criteria. Associations between TFAM and functional outcomes were analyzed using multivariate linear and logistic regressions adjusted for age, sex, comorbidity, and sedentary time. **Results**: Participants in the (Q2–Q4) TFAM quartiles showed a 1.15-point increase in FTS-5 scores (95% CI: 0.23–2.06; *p* = 0.014), indicating poorer physical performance compared to Q1. Weaker balance, slower gait speed, and reduced grip strength were also found. In women, higher TFAM concentrations were strongly related to worse physical function (β = 2.16; 95% CI: 0.89–3.44; *p* = 0.001) and greater impairment across strength-related subcomponents. No significant associations were identified in men. Elevated TFAM was also associated with greater sarcopenia risk in the total sample (OR = 1.56; 95% CI: 1.05–2.31; *p* = 0.028), although sex-stratified analyses were not significant. **Conclusions**: Higher circulating TFAM is associated with poorer functional status, especially in women, and with greater sarcopenia risk, suggesting TFAM as a potential biomarker of age-related musculoskeletal impairment.

## 1. Introduction

Aging entails complex changes at multiple levels, including cumulative disease burden and progressive decline in functional abilities. Functional performance, a key predictor of quality of life, independence, hospitalization risk, long-term care, and mortality, is central to healthy aging [1]. The World Health Organization emphasizes functional ability as a cornerstone of healthy aging, highlighting the challenge of preserving independence and quality of life [2,3]. Skeletal muscle, a metabolically active organ, plays a central role in this context, as it underpins mobility, metabolism, and resilience [4], making it a key determinant of functional status in older adults. With aging, the progressive loss of muscle mass, strength, and function, known as sarcopenia, emerges as a major biological substrate of frailty and contributes to functional decline, increased morbidity and mortality [5]. Affecting 10–27% of community-dwelling older men and women [6], sarcopenia arises from a combination of inactivity and molecular, cellular, hormonal, neurological, and nutritional factors [4]. The pattern and magnitude of age-related muscle loss differ between sexes, with males typically experiencing greater overall muscle mass loss, while females may face earlier strength declines and greater reductions in muscle quality [7,8,9]. Such differences underscore the importance of incorporating a sex-specific perspective when examining sarcopenia and functional impairment related to aging.

Accumulating evidence suggests that mitochondrial damage and dysfunction impact muscle function [10]. It has been proposed that impaired mitochondria increase reactive oxygen species production and deplete cellular energy, particularly in skeletal muscle, leading to mobility loss and functional decline in older adults [11]. Therefore, identifying easily accessible biomarkers associated with mitochondrial alterations may offer valuable insights for improving the diagnosis and monitoring of functional decline in older adults.

Among key mitochondrial factors, mitochondrial transcription factor A (TFAM), a member of the high-mobility group box (HMGB) protein family, is a crucial regulator of mitochondrial DNA (mtDNA) maintenance, and mitochondrial biogenesis [12]. TFAM expression has been reported to decline with aging [13,14], although this pattern seems to be tissue-specific [13]. Decreased expression of TFAM in aged muscle has been linked to impaired mitochondrial function, increased oxidative stress, and the loss of muscular strength and mass seen in sarcopenia [15]. Moreover, TFAM upregulation through exercise and pharmacological treatments have been proposed as potential interventions to prevent muscle atrophy by enhancing mitochondrial adaptations and function [16].

Beyond its role within the mitochondria, TFAM can be released into the extracellular space when mitochondria are damaged, acting as a mitochondria-derived damage-associated molecular pattern (DAMP), triggering a systemic inflammatory response [17]. Other DAMPS, such as circulating cell-free DNA (ccf-DNA), particularly when derived from mitochondrial DNA (ccf-mtDNA), have been identified as potential markers of chronic inflammation and cellular stress in some age-related conditions [18,19]. This release makes TFAM a promising circulating biomarker for early detection and targeted intervention in age-related muscle alterations. Interestingly, a recent study on the VIVIFRAIL exercise protocol reported that significant functional improvements in older adults were accompanied by a reduction in TFAM mRNA expression in peripheral blood mononuclear cells (PBMCs), suggesting a link between exercise, TFAM, and functional outcomes [20].

However, the relationship between circulating TFAM levels, sarcopenia and functional impairment remains poorly addressed, particularly in a sex-specific context. Addressing this question is critical because of the sex-based differences in muscle aging.

Taken as a whole, this study aims to investigate the association between circulating TFAM levels, sarcopenia, and physical function in older adults from the Toledo Study of Healthy Aging (TSHA), with a focus on potential sex-specific differences.

## 2. Materials and Methods

### 2.1. Study Participants

This cross-sectional study used baseline data from the second wave of the TSHA, a Spanish cohort designed to examine cognitive and functional determinants in community-dwelling adults aged 65 years or older from the province of Toledo, Spain. A total of 989 older subjects with complete baseline sociodemographic and clinical data were initially included in the study. However, the sample size for specific outcomes, including physical performance tests (FTS-5, Romberg, gait speed, etc.) and sarcopenia assessment, varied slightly due to missing values. These discrepancies arose because some participants were unable to complete every functional assessment due to physical limitations and post hoc database cleaning. We followed a complete-case analysis approach for each specific outcome, whereby only participants with available data for that particular outcome were included to maximize the statistical power of each individual analysis.

The TSHA cohort follows standardized procedures that have been previously described [21]. Plasma samples were stored at −80 °C within a biobank facility. To ensure sample integrity, they were transferred to the testing site only at the time of analysis, strictly adhering to established safety and quality protocols.

Anthropometric measurements, including height and weight, were recorded at baseline to calculate the Body Mass Index (BMI) (kg/m^2^).

### 2.2. Study Variables

#### 2.2.1. TFAM Levels

Plasma TFAM levels were determined in plasma from the 989 participants using a competitive enzyme-linked immunosorbent assay kit (EH12912, FineTest, Wuhan, China).

These kits are based on sandwich ELISA technology. The capture antibody is pre-coated onto 96-well plates, and a biotin-conjugated antibody is used as the detection antibody. Standards, samples (diluted 1:4 with the appropriate diluent from the kit), and the biotin-conjugated detection antibody are then added to the wells. Horseradish peroxidase (HRP)-streptavidin is added, and unbound conjugates are washed off with wash buffer. Tetramethylbenzidine (TMB) substrates are used to visualize the HRP enzymatic reaction, with the TMB being catalyzed by HRP to produce a blue product that turns yellow after the addition of an acidic stop solution. The intensity of the yellow color is dependent on the amount of TFAM in each sample loaded onto the plate. The absorbance is read at 450 nm using a microplate reader (Spectra Max Plus; Molecular Devices, San José, CA, USA) and the sample concentration is calculated by interpolating the signal on the calibration curve. All samples were analyzed in duplicate according to the manufacturer’s instructions.

#### 2.2.2. Frailty Trait Scale (FTS-5)

Functional performance was assessed using the FTS-5, following the methodology described by García-García et al. [22]. The instrument evaluates five domains: 1-energy balance and nutrition (estimated through body mass index, BMI), 2-physical activity (Physical Activity Scale for the Elderly, PASE), 3-nervous system function (Romberg test), 4-muscle strength (handgrip dynamometry), and 5-gait speed (3 m walking test). Each item is scored from 0 (best performance) to 10 (worst performance).

#### 2.2.3. Sarcopenia

Sarcopenia was defined according to the criteria established by the Foundation for the National Institutes of Health (FNIH) [23], adapted for the TSHA (standardized FNIH, sFNIH) [24], as follows: (i) low isometric handgrip strength (IHG) (<25.51 kg for men and <19.19 kg for women), (ii) low muscle mass, based on appendicular lean soft mass (aLM) (<0.65 in men and <0.54 in women), and (iii) low gait speed (<0.8 m/s). Participants were classified as sarcopenic only when they met all three sFNIH criteria.

IHG was assessed using a hydraulic Jamar dynamometer (J. A. Preston Corporation, Clifton, NJ, USA), according to standard procedures. The highest value (in kilograms) of three trials was used for analysis. One minute rest interval was provided among attempts. aLM was measured through a whole-body dual-energy X-ray absorptiometry (DXA) on a Hologic scanner (Bedford, MA, USA). Body weight and height were determined using a stadiometer and an analog medical scale, respectively. BMI was estimated as body weight in kilograms (adjusted to the nearest 0.1) divided by height (adjusted to the nearest cm) in square meters. Then, aLM adjusted by BMI (aLM/BMI) was obtained. For the gait speed assessment, participants were instructed to walk 3 m at their usual pace. The fastest of two trials (measured in meters per second) was analyzed.

#### 2.2.4. Adjusting Variables

Adjusting variables were selected based on prior evidence and biological plausibility as potential confounders. These included: age, sex, comorbidity burden (assessed by the Charlson index [25] and sedentary behavior. Sedentary behavior, measured as the amount of weekly sitting time obtained from the PASE questionnaire, was included as a covariate, considering its established association with poorer physical function and its potential influence on mitochondrial health and physical capacity [26].

### 2.3. Statistical Analysis

Continuous variables were presented as mean ±standard deviation (SD) and categorical variables as number and percentage (N, %). Plasma TFAM concentrations were reported as geometric mean ± geometric SD factor. Differences between groups were tested using Mann–Whitney and Chi-square test, respectively.

Associations between TFAM concentrations and functional impairment were assessed using multivariate linear and logistic regression model adjusted for age, sex, Charlson index and sedentary time.

Given the observed distribution of TFAM, the results for the FTS-5 scale, its individual subcomponents and sarcopenia were presented by comparing quartiles Q2–Q4 with the first quartile Q1 (reference category).

Statistical analyses were conducted using R for Windows (Vienna, Austria) version 4.5.1., with *p* value < 0.05 considered statistically significant.

## 3. Results

### 3.1. Characteristics of Participants from TSHA

Characteristics of study participants are summarized in Table 1. A total of 989 individuals (mean age: 75.4 years) were included. Participants in Q1 were slightly but significantly younger than those in Q2–Q4 (74.9 ± 6.6 vs. 75.6 ± 5.8 years, *p* = 0.024). Men comprised 45.6% of the sample, with no differences across groups.

No significant variations were observed between quartiles in BMI, weekly sitting time, Charlson Index, or number of prescribed medications. Polypharmacy prevalence was 56.6%, with a comparable distribution between Q1 and Q2–Q4.

The overall sarcopenia prevalence was 26.8%, with a lower frequency in Q1 than Q2–Q4 (21.6% vs. 28.5%; *p* = 0.039). Functional performance, assessed by the FTS-5, was poorer in Q2–Q4 (16.9 ± 7.5 vs. 15.5 ± 6.7; *p* = 0.012). No differences were found in Katz Index scores or the prevalence of disability in basic activities of daily living (BADL) (Table 1).

A sex-stratified analysis further explored associations between circulating TFAM, functional deterioration, and sarcopenia (Table 2).

**Figure 1:**
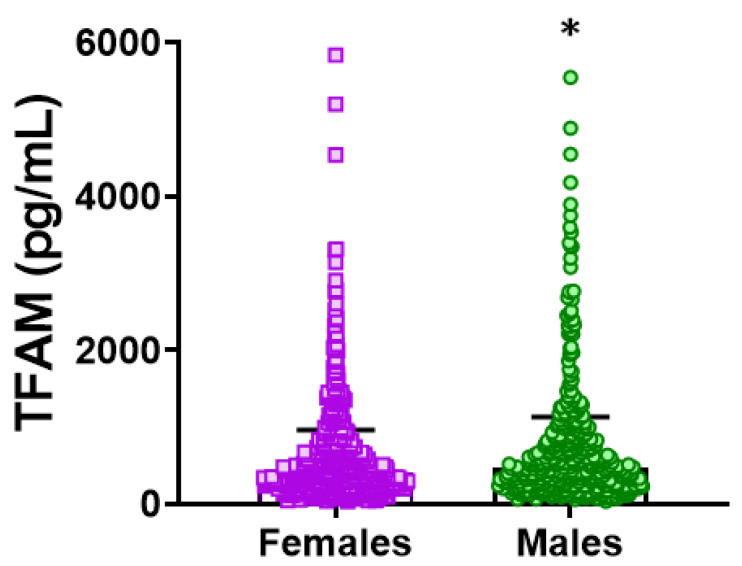
Circulating TFAM levels stratified by sex. Scatter dot plot showing the distribution of plasma TFAM concentrations (pg/mL) in women (*n* = 538) and men (*n* = 451). Each dot represents an individual value. The horizontal line and error bars indicate the geometric mean and geometric standard deviation, respectively. * *p* < 0.05 by Mann–Whitney U test. TFAM: mitochondrial transcription factor A.

Among women (*n* = 538; mean age: 75.5 years), no significant age differences existed across TFAM quartiles. Women had lower circulating TFAM concentrations than men (mean TFAM 405.6 ± 2.4 vs. 465.9 ± 2.4, *p* = 0.024), as illustrated in Figure 1. Women in Q1 (mean TFAM ≤ 136.9 pg/mL, *n* = 135) showed no differences in BMI, comorbidity, or medication use compared with Q2–Q4 (mean TFAM ≥ 583.6 pg/mL, *n* = 403).

Sarcopenia prevalence tended to be lower in Q1 compared with Q2–Q4 (32.8% vs. 41.7%) without reaching statistical significance (*p* = 0.07). Women in Q1 displayed better physical performance, with lower FTS-5 scores (16.6 vs. 19.1; *p* = 0.001) similar to that observed in the whole sample. Katz Index scores and BADL disability showed no significant differences (Table 2).

Among men (*n* = 451; mean age 75.2 ± 6.0 years), Q1 participants were younger than those in Q2–Q4 (74.2 ± 6.0 vs. 75.6 ± 5.9 years; *p* = 0.023). Consistent with the overall findings, no significant differences were found in BMI, comorbidity, nor medication. Sarcopenia prevalence was higher among men with elevated TFAM levels, but FTS-5 score and BADL dependency did not differ (Table 2).

### 3.2. Higher Circulating TFAM Levels Are Associated with Impaired Functional Status and Muscle Strength-Related Performance in Older Subjects

Participants in upper TFAM quartiles exhibited significantly higher FTS-5 scores (Table 1), indicating poorer physical function. Analysis of circulating TFAM in relation to functional status showed a consistent pattern. Individuals with higher TFAM levels, compared with those in the lowest quartile, demonstrated poorer performance on the FTS-5 and its muscle strength-related subcomponents. Specifically, higher TFAM was associated with a 1.15-point increase in FTS-5 scores (95% CI: 0.23–2.06; *p* = 0.014), reflecting worse overall functional capacity (Table 3). Similarly, higher TFAM was linked to poorer performance in other functional domains, with higher β values reflecting greater impairment, including reduced balance (Romberg test: β = 0.45; 95% CI: 0.10–0.81; *p* = 0.012), slower gait speed (β = 0.61; 95% CI: 0.24–0.98; *p* = 0.001), and diminished grip strength (β = 0.29; 95% CI: 0.03–0.56; *p* = 0.032). Collectively, these associations suggest a potential link between circulating TFAM and muscle function deterioration.

### 3.3. Sex-Stratified Analysis of the Association Between TFAM Levels and Impaired Functional Status

In women, the relationship between circulating TFAM levels and physical performance mirrored the overall study patterns. Consistent with the poorer functional outcomes seen among those in higher quartiles compared with the lowest (Table 2), higher TFAM concentrations were significantly associated with greater functional deterioration (Table 4). For FTS-5 scores, the β coefficient was 2.16 (95% CI: 0.89–3.44; *p* = 0.001). Comparable associations were observed for the muscle strength-related subcomponents of the FTS-5. Specifically, higher TFAM was associated with poorer balance on the Romberg test (β = 0.65; 95% CI: 0.15–1.14; *p* = 0.010), slower gait speed (β = 0.68; 95% CI: 0.17–1.19; *p* = 0.009), and reduced grip strength (β = 0.38; 95% CI: 0.01–0.76; *p* = 0.045). Collectively, these findings reinforce the idea that elevated TFAM is significantly associated with impaired physical function in women, paralleling the associations detected in the total study cohort, although formal TFAM × sex interaction analyses were not statistically significant.

In contrast, circulating TFAM levels in men (where FTS-5 scores showed no significant quartile differences (Table 2)) were not associated with functional performance (Table 4). The β coefficient for TFAM with FTS-5 scores was -0.16 (95% CI: −1.46 to 1.14; *p* = 0.809). Similarly, no significant associations were observed between TFAM and balance (β = 0.21; 95% CI: −0.29 to 0.71; *p* = 0.417), gait speed (β = 0.52; 95% CI: −0.02 to 1.05; *p* = 0.058), or grip strength (β = 0.17; 95% CI: −0.21 to 0.56; *p* = 0.377). Thus, unlike in women, elevated TFAM does not appear to influence physical function in men.

### 3.4. Association Between TFAM and Sarcopenia

As previously shown, circulating TFAM levels are associated with poorer muscle-related outcomes, including balance, grip strength, and gait speed, both overall and in women. Based on these observations, we further evaluated the relationship between TFAM and sarcopenia. In the total population (*n* = 945), higher TFAM was significantly associated with 56% higher odds of sarcopenia (OR = 1.56; 95% CI: 1.05–2.31; *p* = 0.028) (Table 5).

Sex-stratified analyses showed non-significant association in women (*n* = 512; OR = 1.45; 95% CI: 0.93–2.26; *p* = 0.101) and men (*n* = 433; OR = 2.02; 95% CI: 0.84–4.88; *p* = 0.117). Although the effect sizes were consistent with the overall association, these results did not reach statistical significance in sex-specific analyses, potentially reflecting reduced statistical power in the stratified subgroups. In addition, formal TFAM × sex interaction analyses were not statistically significant.

## 4. Discussion

This study demonstrates for the first time that elevated plasma TFAM levels are consistently associated with poorer functional performance, more evident in women, and higher sarcopenia risk in a population-based cohort of older adults.

TFAM is considered a guardian of mitochondrial integrity and function [12]. Mitochondrial dysfunction is widely recognized as a hallmark of pre-frailty and early muscle decline in the elderly [27]. Reduced muscle TFAM expression has been linked to functional deterioration in animal models [14], highlighting the relevance of preserved mitochondrial function for maintaining physical capacity. In human aging, TFAM expression shows complex, tissue-specific changes, with some studies reporting a marked increase with age [28].

When released into the cytosol or circulation due to mitochondrial damage, TFAM acts as a DAMP, inducing inflammatory responses [29], making DAMPs potential markers of mitochondrial stress. Recent findings indicate a potential link between mitochondrial dysfunction and low-grade inflammation mediated by DAMPs, including TFAM among others [30], both hallmarks of aging and drivers of functional decline [31]. Other circulating DAMPs such as cell-free mitochondrial DNA sources have been longitudinally associated with different physical outcomes among community dwelling older adults [18]. However, no studies have examined the relationship between circulating TFAM levels and aging-related functional impairment.

The present study identifies mitochondrial stress as a potential factor associated with age-related functional impairment. In the TSHA cohort, higher circulating TFAM levels were associated with poorer functional performance, as participants in upper TFAM quartiles presented significantly higher FTS-5 scores. Notably, no major differences across quartiles were observed in BMI, comorbidity burden, or polypharmacy, suggesting an independent association between TFAM and physical impairment. Therefore, circulating TFAM may represent a valuable marker of mitochondrial dysfunction for diagnosing and monitoring functional decline in older adults. However, the lack of differences in BADL disability indicates that the functional impact of TFAM is likely limited to early or subclinical stages, rather than overt dependency.

Given known sex differences in both functional decline prevalence and underlying mechanisms with women manifesting higher rates of pre-frailty and frailty [32], it is essential to consider the biological factors contributing to these disparities. Mitochondrial aging also differs between men and women, likely due to sex-specific factors such as hormonal influences [33], which may increase women’s susceptibility to age-related functional decline. Accordingly, sex-stratified analyses revealed distinct patterns. In women, elevated TFAM levels were strongly and significantly associated with functional deterioration, as those in the higher TFAM quartiles consistently showed elevated FTS-5 scores, with stronger effect sizes than in the overall cohort. Women in the lowest TFAM quartile showed better physical performance, supporting the robustness of this association. In contrast, despite higher TFAM concentrations in men, no significant associations with FTS-5 or other functional measures were observed. Interestingly, although men in higher TFAM quartiles were older, functional status did not differ, further highlighting women’s possibly greater sensitivity to functional derangements. Collectively, these findings suggest that elevated circulating TFAM is a potential marker of impaired functional health in older adults, which seems to be more evident in women in sex-stratified analyses, although formal interaction tests by sex were not significant.

Age-related functional decline is often underpinned by skeletal muscle deterioration known as sarcopenia [34]. Mitochondrial damage and dysfunction are well recognized as key contributors to muscle performance decline with aging [11,35]. In the present study, higher TFAM levels were associated with a 56% increase in the odds of sarcopenia in the overall population, suggesting that systemic TFAM elevations may serve as a biological indicator or be closely associated with the processes involved in muscle loss. Supporting our findings, a high level of other DAMPs, such as ccf-mtDNA, has been independently associated with sarcopenia and positively correlated with the inflammatory marker IL-6 in older individuals [36]. In addition, the above associations observed in the TSHA cohort extended to specific muscle performance-related domains of the FTS-5 score. Increased TFAM concentrations were related to reduced balance, slower gait speed, and diminished grip strength, which are established markers of frailty and sarcopenia [37], suggesting a potential link between TFAM and muscle function deterioration. In a recent study by Nidadavolu et al., no cross-sectional associations were found between ccf-mtDNA fragments and cognitive or physical outcomes. However, higher levels of long ccf-mtDNA fragments were associated with a decline in composite gait scores over an eight-year period [18]. The observed association between circulating TFAM and sarcopenia in our cohort supports the hypothesis that mitochondrial alterations are associated with muscle function deterioration and suggests that these changes can be monitored at a systemic level.

Importantly, the pattern and extent of skeletal muscle deterioration differ between sexes. Men are generally more susceptible to reductions in muscle mass, whereas women tend to experience earlier and more pronounced impairments in muscle quality and strength [38,39]. In parallel, mitochondrial aging within skeletal muscle also exhibits sex-specific characteristics, with both the onset and severity of dysfunction appearing to be influenced by sex-specific factors [33]. These distinct patterns underscore the need for sex-specific strategies in both frameworks and clinical interventions.

When stratified by sex, this study showed a trend toward higher sarcopenia prevalence in women with elevated TFAM levels, albeit this did not reach statistical significance (*p* = 0.07). Similarly, higher TFAM levels were not significantly associated with increased sarcopenia odds in older women from TSHA, probably reflecting limited statistical power due to smaller sample sizes in stratified analyses. Notably, sarcopenia prevalence was higher in women compared to men, reflecting a greater impact on muscle. Consequently, even modest or statistically non-significant muscle mass loss in women may be sufficient to lead to meaningful declines in physical function, as evidenced by our findings. Accordingly, elevated TFAM was significantly correlated with deteriorations in muscle-related functions, including poorer balance, slower gait speed and reduced grip strength.

In men, those in the higher TFAM quartiles exhibited a greater prevalence of sarcopenia compared to the lowest quartile, though this association was not statistically significant despite a positive trend (OR = 2.02). Likewise, no significant associations were observed between TFAM and muscle performance measures such as balance, grip strength, or gait speed. The absence of associations in men raises the possibility of sex-mediated protective mechanisms, differences in muscle composition, or regulatory pathways affecting TFAM’s role in addition to the limited sample size within the male subgroup. These findings underscore the need to consider sex-specific biological and molecular factors when investigating sarcopenia and functional decline in aging populations.

Collectively, these findings suggest that elevated circulating TFAM may serve as a biomarker of impaired muscle and functional health in older adults, which is more evident in women. From a mechanistic perspective, increased plasma TFAM may reflect a physiological response to mitochondrial alteration, whereby stressed or damaged mitochondria export TFAM into the circulation as part of disrupted quality control mechanisms. This phenomenon could signal ongoing mitochondrial damage in skeletal muscle, contributing to diminished muscle strength and function leading to age-related functional decline. However, beyond its role as a putative systemic marker, circulating TFAM might exert inflammatory effects, as suggested by previous studies highlighting the role of mitochondrial DAMPs in mediating inflammatory pathways. Specifically, mitochondrial perturbations can activate the NLRP3 inflammasome complex, triggering inflammatory signaling cascades that adversely impact muscle quality control mechanisms [40]. Moreover, dysregulation of toll-like receptors (TLRs) and the NLRP3 inflammasome pathways have been linked to frailty and sarcopenia [41]. Nevertheless, since inflammatory markers and skeletal muscle mitochondrial function were not directly assessed in the present study, these mechanisms should be considered speculative and based on the previous literature, rather than conclusions directly supported by our data. Thus, elevated circulating TFAM might both reflect mitochondrial stress and participate in the activation of inflammatory processes, potentially contributing to muscle deterioration during aging. Consequently, it would be valuable for future research to evaluate circulating TFAM alongside inflammatory markers, such as C-reactive protein (CRP) and interleukin-6 (IL-6). Given that mitochondrial stress and chronic low-grade inflammation are closely interrelated in aging and sarcopenia, their integration could provide a more comprehensive and accurate biological interpretation of mitochondrial homeostasis in older adults. Ultimately, longitudinal and experimental studies are needed to clarify the directionality and nature of these complex relationships.

Although this study offers valuable insights, certain limitations should be acknowledged. Its cross-sectional design precludes causal inference, and the limited sample size for sex-stratified analyses may have reduced the power to detect moderate effects in men. Despite these constraints, the use of a well-characterized community-based cohort and comprehensive evaluation of both clinical and biochemical endpoints strengthen the validity and enhance the generalizability of the findings. Furthermore, because mitochondrial function is difficult to assess directly in clinical practice, identifying TFAM as a potential circulating biomarker offers a practical alternative for earlier detection and more precise monitoring of functional decline and sarcopenia in aging populations, potentially enabling more personalized interventions for older adults who are at the highest risk.

## 5. Conclusions and Future Perspectives

In conclusion, higher circulating TFAM levels are strongly associated with impaired functional status, particularly in women, and with an increased risk of sarcopenia in older adults, supporting the potential biological link between mitochondrial dysfunction and muscle impairment. Although these associations were mainly observed in women in sex-stratified analyses, formal interaction tests by sex were not statistically significant. These findings emphasize the potential of TFAM as a useful biomarker that can improve diagnostic accuracy for interventions aimed at preventing or delaying age-related musculoskeletal decline.

Regarding future perspectives, it is important to note that higher circulating TFAM is not necessarily detrimental, and its significance likely depends on the specific biological context. Consequently, findings related to circulating TFAM should not be directly extrapolated to other tissues or physiological systems. While our data highlights an association between higher TFAM levels (Q2–Q4 vs. Q1) and functional decline, this association may be non-linear, suggesting that both abnormally high and low TFAM concentrations could reflect impaired mitochondrial homeostasis. Furthermore, since circulating TFAM does not currently represent a direct therapeutic target, it should be regarded as a potential biomarker of the mitochondrial state and systemic bioenergetic stress closely associated with functional impairment and sarcopenia.

## Figures and Tables

**Table 1:** Characteristics of participants from the Toledo Study of Healthy Aging. Data are compared to TFAM levels in the first quartile.

	All	Q1	Q2–Q4	*p*-Value
Number	989	247	742	
Age, years	75.41 (5.99)	74.86 (6.62)	75.59 (5.76)	**0.024**
Sex, men	451 (45.60)	107 (43.32)	344 (46.36)	0.406
BMI (kg/m^2^)	29.34 (4.72)	29.20 (4.68)	29.38 (4.74)	0.569
Sitting time (h/week)	22.28 (8.26)	21.60 (7.97)	22.51 (8.35)	0.059
Charlson Index	1.34 (1.68)	1.47 (1.70)	1.30 (1.67)	0.076
Number of medications	5.16 (2.89)	5.38 (2.99)	5.09 (2.85)	0.224
Polypharmacy	560 (56.62)	143 (57.89)	417 (56.20)	0.642
Sarcopenia	253 (26.77)	51 (21.61)	202 (28.49)	**0.039**
FTS-5 score	16.56 (7.33)	15.45 (6.67)	16.93 (7.51)	**0.012**
Katz Index score	5.73 (0.69)	5.79 (0.52)	5.71 (0.73)	0.258
Disability for BADL	191 (19.43)	42 (17.07)	149 (20.22)	0.281
TFAM (pg/mL)	432.06 (2.40)	144.39 (1.51)	622.30 (1.93)	**0.000**

Numerical variables are expressed as mean± standard deviation (SD) while discrete variables are expressed as number and (percentage). Cutoff point of TFAM between Q1 and Q2 was 241.17 pg/mL. TFAM concentrations are expressed as geometric mean ± geometric SD factor. BADL: basic activities for daily living; BMI: body mass index; FTS-5: frailty trait scale-5; TFAM: mitochondrial transcription factor A. Significant associations are highlighted in bold.

**Table 2:** Characteristics of female and male participants from the Toledo Study of Healthy Aging. Data are compared to TFAM levels in the first quartile.

	WOMEN				MEN			
	All	Q1	Q2–Q4	*p*-Value	All	Q1	Q2–Q4	*p*-Value
Number	538	135	403		451	113	338	
Age (years)	75.54 (6.01)	75.45 (7.02)	75.57 (5.64)	0.350	75.24 (5.98)	74.19 (6.01)	75.59 (5.94)	**0.023**
BMI (kg/m^2^)	30.05 (5.09)	29.35 (4.65)	30.28 (5.21)	0.069	28.49 (4.08)	28.89 (4.76)	28.35 (3.83)	0.413
Sitting time (h/week)	23.05 (8.05)	22.73 (7.36)	23.16 (8.28)	0.316	21.36 (8.42)	20.50 (8.47)	21.65 (8.40)	0.159
Charlson Index	1.34 (1.60)	1.47 (1.67)	1.30 (1.58)	0.259	1.34 (1.76)	1.47 (1.75)	1.30 (1.77)	0.162
No. of medications	5.53 (2.81)	5.53 (3.03)	5.53 (2.73)	0.878	4.71 (2.92)	5.00 (2.99)	4.62 (2.89)	0.258
Polypharmacy	336 (62.45)	61.48	62.78	0.788	224 (49.67)	50.44	49.41	0.849
Sarcopenia	202 (39.45)	32.81	41.67	0.076	51 (11.78)	6.42	13.58	**0.045**
FTS-5 score	18.43 (7.25)	16.58 (6.91)	19.05 (7.26)	**0.001**	14.36 (6.81)	13.82 (5.92)	14.54 (7.09)	0.606
Katz Index score	5.67 (0.75)	5.73 (0.60)	5.65 (0.80)	0.415	5.80 (0.59)	5.85 (0.41)	5.78 (0.64)	0.566
Disability for BADL	124 (23.09)	20.74	23.88	0.454	67 (15.02)	13.39	15.57	0.577
TFAM (pg/mL)	405.59 (2.37)	136.90 (1.54)	583.58 (1.89)	**<0.001**	465.90 (2.42)	154.73 (1.46)	673.49 (1.96)	**<0.001**

Numerical variables are expressed as mean ± standard deviation (SD), while discrete variables are expressed as number and (percentage). Cutoff points of TFAM between Q1 and Q2 were 234.47 pg/mL and 245.97 pg/mL for women and men, respectively. TFAM concentrations are expressed as geometric mean ± SD factor. BADL: basic activities for daily living; BMI: body mass index; FTS-5: frailty trait scale-5; TFAM: mitochondrial transcription factor A. Significant associations are highlighted in bold.

**Table 3:** Increased TFAM levels are related to functional impairment among participants from Toledo Study of Healthy Aging.

	Number	TFAM Beta (95%, CI)	*p* Value
FTS-5	934	1.15 (0.23, 2.06)	**0.014**
Romberg	949	0.45 (0.10, 0.81)	**0.012**
Gait speed	950	0.61 (0.24, 0.98)	**0.001**
Grip strength	988	0.29 (0.03, 0.56)	**0.032**

Data are expressed as beta coefficients with 95% confidence intervals, comparing TFAM quartiles (Q2–Q4) against the lowest quartile (Q1). Associations between TFAM and the different outcomes were adjusted for age, sex, Charlson index and the weekly sitting time. Positive beta values for each outcome indicates poorer function. Significant associations are highlighted in bold.

**Table 4:** Elevated circulating TFAM is associated with functional decline in women, but not in men, in the Toledo Study of Healthy Aging.

	Number	Beta (95%, CI)	*p* Value
**Women**			
FTS-5	505	2.16 (0.89, 3.44)	**0.001**
Romberg	515	0.65 (0.15, 1.14)	**0.010**
Gait speed	516	0.68 (0.17, 1.19)	**0.009**
Grip strength	537	0.38 (0.01, 0.76)	**0.045**
**Men**			
FTS-5	429	−0.16 (−1.46, 1.14)	0.809
Romberg	434	0.21 (−0.29, 0.71)	0.417
Gait speed	434	0.52 (−0.02, 1.05)	0.058
Grip strength	451	0.17 (−0.21, 0.56)	0.377

Data are expressed as beta coefficients with 95% confidence intervals, comparing TFAM quartiles (Q2–Q4) against the lowest quartile (Q1). Association between TFAM and the different outcomes were adjusted for age, Charlson index and the weekly sitting time. Positive beta values for each outcome indicates poorer function. Significant associations are highlighted in bold.

**Table 5:** Elevated circulating TFAM is associated with sarcopenia in participants from the Toledo Study of Healthy Aging.

	Number	OR (95%, CI)	*p* Value
**Sarcopenia**			
All participants	945	1.56 (1.05, 2.31)	**0.028**
Women	512	1.45 (0.93, 2.26)	0.101
Men	433	2.02 (0.84, 4.88)	0.117

Data are expressed as odds ratio (OR) with 95% confidence intervals comparing TFAM quartiles (Q2–Q4) against the lowest quartile (Q1). Association between TFAM and sarcopenia were adjusted for age, sex, Charlson index and weekly sitting time. Significant associations are highlighted in bold.

## Data Availability

The data presented in this study are available on request from the corresponding author due to ethical restrictions.

## Abbreviations

The following abbreviations are used in this manuscript:

BADL	Basic activities for daily living
ccf-DNA	Circulating cell-free DNA
ccf-mtDNA	Circulating cell-free mitochondrial DNA
CI	Confidence interval
DAMP	Damage-associated molecular patterns
ELISA	Enzyme-linked immunosorbent assay
FNIH	Foundation for the National Institutes of Health
FTS-5	Frailty trait scale-5
HMGB	High-mobility group box
mtDNA	Mitochondrial DNA
OR	Odd ratio
PASE	Physical activity scale for the elderly
PBMCs	Peripheral blood mononuclear cells
SD	Standard deviation
sFNIH	Standardized FNIH
TFAM	Mitochondrial transcription factor A
TSHA	Toledo Study of Healthy Aging
WHO	World Health Organization
